# MicroRNA-129-5p alleviates spinal cord injury in mice via suppressing the apoptosis and inflammatory response through HMGB1/TLR4/NF-κB pathway

**DOI:** 10.1042/BSR20193315

**Published:** 2020-03-12

**Authors:** Guang Wan, Yongbo An, Jingang Tao, Yanli Wang, Qinglan Zhou, Rongli Yang, Qiudong Liang

**Affiliations:** 1 Department of Orthopedics, The First Affiliated Hospital of Xinxiang Medical University, Weihui 453100, Henan, China; 2 Operation Room,The First Affiliated Hospital of Xinxiang Medical University, Weihui 453100, Henan, China

**Keywords:** HMGB1//TLR4/NF-κB pathway, inflammatory response, miR-129-5p, Spinal cord injury

## Abstract

Secondary injury after spinal cord injury (SCI) is one reversible pathological change mainly involving excessive inflammatory response and neuro-apoptosis. Since in recent years, microRNAs (miRNAs) have been proposed as novel regulators of inflammation in different disease conditions. However, the role of miRNAs in the inflammatory response and apoptosis of secondary injury after SCI remains to be fully elucidated. Here, we tried to explore the influence and mechanism of miRNAs on the neuron inflammatory response and apoptosis after SCI. The expression profiles of miRNA were examined using miRNA microarray, and among the candidate miRNAs, miR-129-5p was found to be the most down-regulated miRNA in spinal tissues. Overexpression of miR-129-5p using agomir-miR-129-5p promoted injury mice functional recovery, suppressed the apoptosis and alleviated inflammatory response in spinal tissues. Using LPS-induced BV-2 cell model, we found miR-129-5p was also proved in protecting inflammatory response and cell apoptosis *in vitro*. High-mobility group protein B1 (HMGB1), a well-known inflammatory mediator, was found to be directly targeted by miR-129-5p and it was associated with the inhibitory effect of miR-129-5p on the activation of toll-like receptor (TLR)-4 (TLR4)/ nuclear factor-κB (NF-κB) pathway *in vitro* and *in vivo*. Further experiments revealed that the anti-apoptosis and anti-inflammatory effects of miR-129-5p were reversed by HMGB1 overexpression in BV-2 cells. Collectively, these data revealed that miR-129-5p alleviated SCI in mice via suppressing the apoptosis and inflammatory response through HMGB1//TLR4/NF-κB pathway. Our data suggest that up-regulation of miR-129-5p may be a novel therapeutic target for SCI.

## Introduction

Spinal cord injury (SCI) is a traumatic injury that results in permanent impairment of the strength in the parts of the body served by the spinal cord below the levels of the injury. The annual incidence rate of SCI are still ∼23 cases per million every year [1]. Although many therapies have been explored for the improvement of patients with SCI, all demonstrated limited efficacy thus far. SCI is characterized by primary and secondary injuries. The former is irreversible physical injury to the spine, whereas the latter is a series of chemical, reversible secondary pathophysiological changes including neuro-inflammatory response, neuronal apoptosis, and oxidative stress, worsening neurologic recovery [2–4]. Secondary injury is reversible and can be controlled. Therefore, the alleviation of neuro-inflammatory response and apoptosis can be an effective therapeutic intervention in secondary injury after SCI.

Excessive inflammatory response is a major risk factor for secondary injury after SCI [5], which can release large amounts of pro-inflammatory cytokines that ultimately result in neuronal cell death [6–11]. Previous studies have indicated that excessive inflammatory response can promote apoptosis and further aggravate secondary injury [12,13]. However, the factors that transmit inflammatory signals after SCI have not been elucidated in detail. Currently, nuclear factor-κB (NF-κB) is considered as an important transcription factor of inflammatory mediators, and has been implicated in the pathophysiology of secondary SCI [14,15]. After SCI, NF-κB is usually activated in nerve cells and microglia and promoted the secretions of inflammatory cytokines, leading to the enlargement of injury area and the damage of anatomic structures [16,17]. High-mobility group box 1 (HMGB1) is a well-known regulator in inflammatory responses. It has previously been reported that HMGB1 participates in the pathogenesis of SCI by triggering toll-like receptor (TLR)-4 (TLR4) and adaptor protein MyD88, subsequently leading to activation of NF-κB signaling and resulting in pro-inflammatory cytokines production [18]. Therefore, the suppression of the HMGB1/TLR4/NF-κB activation may be an effective strategy for treatment of SCI.

MicroRNAs (miRNAs) are single-stranded non-coding RNAs (∼18–25 nucleotides in length), which typically inhibit the translation and target the degradation of mRNAs through partial complementarity [19,20]. Numerous studies have shown the involvement of differently expressed miRNAs in the central nervous system (CNS) injury including SCI [21–23]. For example, Feng et al. [24] showed that miR-204-5p was lowly expressed in the plasma of the SCI patients and overexpression of miR-204 improve SCI through suppressing inflammatory cytokines by targeting SOX11 in SCI mice. Zhu et al. [25] found that overexpressed miR-219-5p promoted SCI recovery and motor function elevation via alleviating NEUROD2-regulated inflammatory response in SCI mice model. However, little studies paid attention to the functions of miRNAs in the pathophysiology of secondary damage in SCI.

In the present study, we analyzed the miRNA profiles in spinal cord tissues from a mouse SCI model. Subsequently, the improvement of neurological dysfunction, inflammatory response, and apoptosis induced by miR-129-5p were verified in mice SCI models. Then, the possible mechanisms of these effects were investigated in LPS-induced BV-2 cell injury model. Our findings suggest that targeting miR-129-5p may be beneficial for preventing secondary SCI.

## Materials and methods

### SCI model establishment

Adult C57BL/6 mice (8- to 10-week-old, weight 20–25 g) were obtained from Shanghai SLAC Laboratory Animal Co., Ltd (China) and housed under standard conditions (12-h light–dark cycle, 25–27°C, ∼40% humidity), and had free access to food and water throughout the duration of the experiments.

Mice were anesthetized with an intraperitoneal (i.p.) injection of 50 mg/kg pentobarbital sodium (Sigma–Aldrich, St. Louis, MO, U.S.A.), followed by laminectomy as previously described [26]. Briefly, an incision was made in the skin, following the medial dorsal line, reaching the aponeurotic and muscular planes, and exposing the posterior vertebral arches from T8 to T12. Under dissection stereomicroscope, 3-mm-long laminectomy, encompassing the caudal end of T10 vertebra and the rostral end of T11 vertebra, was performed. The Infinite Horizons impactor (Infinite Horizons, L.L.C., Lexington, KY, U.S.A.) was used in order to produce the contusion SCI using a force of 60 kdyn/cm^2^. All animal care and experimental procedures were performed at the Animal experimental Center of Xinxiang Medical University and all procedures were approved by the Institutional Animal Care and Use Committee of The First Affiliated Hospital of Xinxiang Medical University. Killing was performed by i.p. injection of sodium pentobarbital (50 mg/kg) followed by cervical dislocation, and mortality was confirmed when there was no spontaneous breathing for 2–3 min and no blinking reflex was observed [27].

### Experimental design

Mice were randomly divided into two groups: SCI and Sham group (*n*=6 each group/time) were subjected to evaluate the locomotor activity using the Basso, Beattie and Bresnahan (BBB) score method at 1, 3, 7, 14, 21, and 28 days after SCI. Sham 7 day, SCI 7 day (*n*=6/group/time) were used to assess spared tissue, apoptosis-related protein expressions using the Cresyl Violet staining and Western blot assay. For microarray analysis and qRT-PCR, the sample size was 3 and 5, respectively.

In another test, mice were randomly divided into four groups to determine the role of agomir-miR-129-5p: SCI group, Sham group, SCI + agomir-miR-129-5p group, and SCI + agomir-negative control (NC) group. Mice were subjected to laminectomy in the SCI group (*n*=6/group/time), while mice that underwent all surgical procedures without injury were used as Sham group. In the SCI + agomir-miR-129-5p group/agomir-NC group (*n*=6/group/time), the mice were subjected to laminectomy and then treated with agomir-miR-129-5p/agomir-NC (50 μl/day, 100 nmol/ml; RiboBio, Guangzhou, China) via intrathecal injection for 3 days (0, 1, and 2 days) starting 15 min after SCI. After 4 weeks, mice were humanely killed with i.p. injection of 50 mg/kg pentobarbital sodium followed by cervical dislocation, and subsequently, the injury spinal cord and serum were harvested for further experiments. The sequences of agomir-miR-129-5p and agomiR-NC are as follows: agomir-miR-129-5p 5′-CUUUUUGCGGUCUGGGCUUGC-3′ (sense), 5′-AAGCCCAGACCGCAAAAAGUU-3′ (antisense) and agomiR-NC 5′-UUCUCCGAACGUGUCACGUTT-3′ (sense), 5′-ACGUGACACGUUCGGAGAATT-3′ (antisense).

### BBB score

BBB score was performed to test locomotor function of mice after SCI as described previously [28], which comprise 21 different criteria for the movement of lower limbs, from complete paralysis to complete mobility. These criteria are based on the accurate observation of the lower limbs, including movement, step, and coordinated motor action. The scores were recorded by two well-trained investigators who were blinded to the experiments.

### Cresyl Violet staining

Cresyl Violet stain was used for staining myelinated axons and the Nissl substance to assess spared myelin volume surrounding the lesion in the injury cord. The spared tissue area was measured using Cresyl Violet staining as described previously [29]. Briefly, spinal cord from indicated groups were fixed with 4% paraformaldehyde (PFA) and embedded in paraffin. Tissue sections (10 μm) were subjected to Cresyl Violet staining, and the lesion was evaluated by ImageJ version 1.46 (Rawak Software, Inc., Munich, Germany).

### Spin al cord water content measurement

The spinal cord tissues obtained in the above experimental procedure at 7 days post-injury were immediately weighted, and then the dry weight of spinal cord tissues was obtained at 70–80°C for 48 h. The ratio of wet-to-dry weight calculated as follows: [(wet weight − dry weight)/wet weight] ×100%.

### Immunohistochemical and immunofluorescent analysis

For immunohistochemical (IHC) analysis, the spinal cord segments were paraffin-embedded and cut into 5-µm-thick slides. Next, the tissue sections were deparaffinized and dehydrated through graded alcohols. The slices were then incubated in 3% H_2_O_2_ for 15 min at room temperature (RT), and then blocked in 10% bovine serum for 30 min. Next, the slices were stained overnight at 4°C with a primary antibody against cleaved caspase-3 (cat. no. 9661, Cell Signaling Technology, Inc, 1:200). Subsequently, the sections were incubated with secondary antibodies (cat. no. 7074; Cell Signaling Technology, Inc. 1:2,00). Finally, the immunoreactivity was visualized by staining with diaminobenzidine (DAB) for 3 min, covered with a coverslip, and analyzed under a light microscope (BX51, Olympus Inc., Tokyo, Japan) at 200× magnification. ImageJ version 1.46 (Rawak Software, Inc., Munich, Germany) was used to quantify IHC staining.

For immunofluorescent analysis (IFA), the BV-2 cells were washed twice with PBS and fixed with 4% paraformaldehyde for 15 min then blocked with 5% BSA for 1 h at RT. Then, the cells were incubated with primary antibodies of cleaved caspase-3 (cat. no. 9661, Cell Signaling Technology, Inc, 1:1000) at 4°C overnight. The next day, after incubation with fluorescent secondary antibodies (Invitrogen) for 1 h, the cells were observed under a fluorescent microscope (Olympus Inc., Tokyo, Japan). ImageJ version 1.46 (Rawak Software, Inc, Munich, Germany) was used to quantify fluorescent staining.

### ELISA

The levels of IFN-α (cat. no. 42120-1), Mouse IL-1β (cat. no. 42400-1), IL-6 (cat. no. D6050) and IL-10 (cat. no.DY417) in mice serum and cell supernatants were measured using ELISA kit (all from R&D Systems, Minneapolis, MN) according to the manufacturer’s protocol. Absorbance was detected using an automatic multi-well spectrophotometer (Bio-Rad Laboratories, Inc., Hercules, CA, U.S.A.) at 450 nm.

### miRNA microarray assay

Total RNA was isolated from spinal cord tissues using RNAEasy plus kit (Qiagen) according to the manufacturer’s protocol. The RNA was quantified by NanoDrop ND-1000 spectrophotometry (Thermo Fisher Scientific, Inc., Waltham, MA, U.S.A.). Total RNA (200 ng) was labeled with fluorescence dye hy3 or hy5 using the miRCURY Hy3/Hy5 Power Labeling kit and hybridized on the miRCURY™ LNA array (v.16.0; Exiqon A/S, Copenhagen, Denmark), which were designed based on miRBaserelease 10.0 and contained 546 probes from humans, mice and rats. The procedure and imaging processes were as described previously [29].

### RNA isolation and quantitative RT-PCR

Total RNA from spinal cord tissues and cells was isolated using TRIzol reagent (TaKaRa, Dalian, China). Reverse transcription of miR-129-5p was synthesized using the miScript II RT kit (Invitrogen, Carlsbad, CA), and cDNA of mRNA was synthesized by using an iScript cDNA synthesis kit (Bio-Rad). For miR-129-5p, the qRT-PCR assays were carried out using an miRNA qPCR detection kit (Tiangen, Beijing). For mRNA, qRT-PCR was conducted by SYBR-Green Gene Expression Assay kit (Tiangen, Beijing) and performed on an ABI Prism 7900 HT (Applied Biosystems). The primers used were as follows: miR-129-5p F 5′-GGGGGCTTTTTGCGGTCTGG-3′, R: 5′-AGTGCGTGTCGTGGAGTC-3′; U6 F: 5′-GCTTCGGCAGCACATATACTAAAAT-3′, R 5′-CGCTTCAGAATTTGCGTGTCAT-3′; HBMG1 F 5′-AGGCTGACAAGGCTCGTTATG-3′, R 5′-TGTCATCCGCAGCAGTGTTG-3′; GAPDH F, 5′-GAAGATGGTGATGGGATTTC-3′, and R, 5′-AACGCTTCACGAATTTGCGT-3′. The relative expression of each gene was calculated using the 2^−∆∆*C*_t_^ method [30].

### Cell culture and treatment

Immortal BV-2 murine microglial cells treated with LPS were widely used to induce cell injuries to mimic the *in vitro* model of SCI [31]. The BV-2 cell line was obtained from ATCC (Manassas, VA, U.S.A.) and maintained in DMEM/F12 (Gibco; Thermo Fisher Scientific, Inc., Waltham, MA, U.S.A.) supplemented with 10% FBS (Gibco), and 1% penicillin and streptomycin (Sigma–Aldrich, St. Louis, MO, U.S.A.) in 5% CO_2_ at 37°C.

BV-2 cells were treated with LPS (Sigma–Aldrich, St. Louis, MO, U.S.A.) at a concentration of 100 ng/ml for 4 h at 37°C as previously reported [32].

### Cell transfection

When BV-2 cells grew to approximately 80% confluence in six-well plate, 20 nM agomiR-129-5p or 2 μg pcDNA-HMGB1 were transfected into cells at 37°C for 48 h, using Lipofectamine® 2000 (Invitrogen). The agomir-miR-129-5p and agomiR NC, were obtained from RiBoBio (Guangzhou, China). HMGB1 overexpressing vector pcDNA-HMGB1 was also constructed by RiboBio (Guangzhou, China).

### Caspase-3 activity

After treatment, the activity of caspase-3 in cell lysates was evaluated using a Caspase-3 Activity Assay kit (Beyotime Institute of Biotechnology), according to the manufacturer’s protocol. The OD value was then measured using a Multiskan Sky microplate reader at an absorbance of 405 nm.

### Luciferase reporter assay

pGL3-HMGB1 wild-type (Wt) or pGL3-HMGB1 mutant type (mut) plasmids were co-transfected with 20 nM agromiR-129-5p into BV-2 cells in 24-well plates (2 × 10^5^/well) using Lipofectamine 2000 reagent (Invitrogen). At 48 h post-transfection, luciferase activity was analyzed using the Dual-Luciferase Reporter Assay system (Promega Corporation) and normalized to *Renilla* luciferase activity.

### Western blot

Western blot was performed as previously described [31]. Briefly, spinal cord tissues and cells were lysed in radio immunoprecipitation assay buffer (Cell Signaling Technology), and protein concentrations were determined by using bicinchoninic acid assays (Cell Signaling Technology). Forty micrograms of extracted protein samples were separated on SDS/PAGE gels and transferred on to a PVDF (Millipore) membrane, and blocked with 5% skimmed milk for 2 h at RT. Then, the membrane was incubated with primary antibodies against HGMB1 (cat. no. 6893), TLR4 (cat. no. 14358), cleaved-caspase-3 (cat. no. 9661), nuclear p-p65 (cat. no. 3033), p65 (cat. no. 8242), Bax (cat. no. 14796), Bcl-2 (cat. no. 4223), cleaved PARP (cat. no. 5625), and β-actin (cat. no. 4970) at 4°C overnight. All antibodies were obtained from Cell Signaling Technology, Inc and the dilution was 1:1000. Subsequently, the blot was incubated with appropriate secondary antibodies (cat. no. 7074; Cell Signaling Technology, Inc. 1:2000) for 1 h at RT. The results were detected using ECL kit (GE Healthcare) and analyzed with ImageJ version 1.46 (Rawak Software, Inc., Munich, Germany).

### Statistical analysis

Statistical analysis was conducted using GraphPad Prism (version 5.0, Inc., La Jolla, CA, U.S.A.). Data were recorded as means  ±  SD. Differences between groups were analyzed using one way ANOVA or Student’s *t* test. A *P*-value <0.05 was considered significant.

## Results

### miR-129-5p was down-regulated in spinal cord of SCI mice

**Figure 1:**
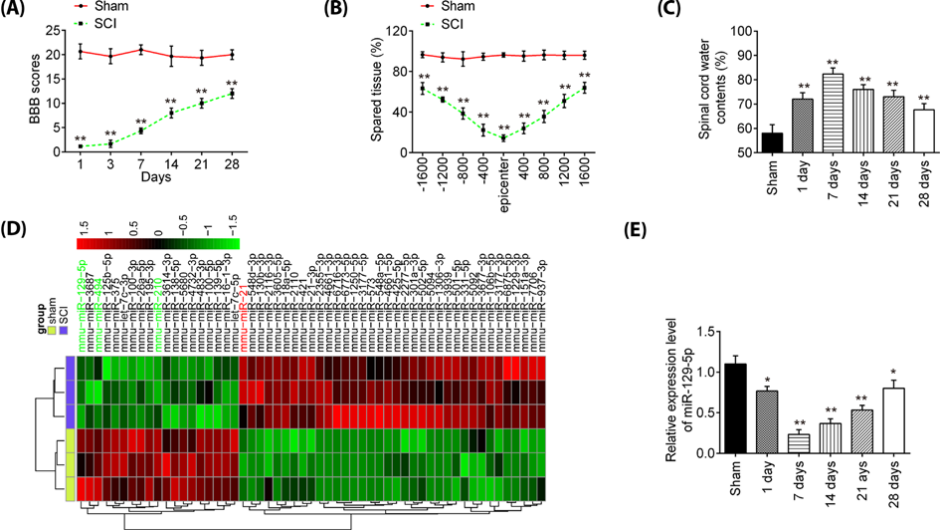
miR-129-5p was down-regulated in spinal cord of SCI rats (**A**) The BBB scores at 1, 3, 7, 14, 21, and 28 days after SCI were shown for all groups of mice. (**B**) Cresyl Violet staining was used to assess spared tissue following behavioral analyses at 28 days post-injury. (**C**) Spinal cord water content was assessed using wet-to-dry weight method. (**D**) Heatmap of normalized expression levels of miRNAs in spinal cord tissues from SCI and sham rats. (**E**) qRT-PCR was performed to determine the expression levels of miR-129-5p in spinal cord tissues from mice at 1, 7, 14, 21, and 28 days after SCI. Data represent the mean ± SD of three independent experiments. *P<0.05, **P<0.01 vs. Sham group.

First, a mice model of SCI injury was established as previously described [26], and then the behavioral analyses of mice were evaluated. As shown in Figure 1A, a spontaneous functional recovery was observed in SCI mice during the experiment, but BBB scores were significantly lower in SCI group than that in the Sham group. Cresyl Violet staining was used to assess spared tissue following behavioral analyses. Sections of thoracic spinal cord were analyzed and the ratio for ‘injured area/total area’ from each section determined. The spared tissue was smaller in SCI mice not only at the injury epicenter, but also in regions extending away from the epicenter, in both rostral and caudal directions, compared with Sham group (Figure 1B). Spinal cord edema was evaluated by detecting water content of spinal cord. It was observed that water contents in spinal cord were significantly increased compared with Sham group and was maximal at 7 days (Figure 1C). All data indicted that SCI model was successfully constructed.

Subsequently, an miRNA array was used to analyze miRNA profiles in SCI tissues; it was found that 19 miRNAs were significantly decreased and 36 miRNAs were increased in SCI group, compared with Sham group (Figure 1D). In our microarray data, miR-494 and miR-210 was decreased while miR-21 was increased, which is consistent with previous reports [29,33,34], suggesting the reliability of our microarray results. But, in our study, miR-129-5p exhibited the most down-regulated alteration in spinal tissues of SCI mice. Notably, a recent study performed by Li et al. [35] reported that miR-129-5p prevented the neuroinflammation and blood–spinal cord barrier damage in spinal cord. However, whether miR-129-5p can exert protective effects against inflammatory response and apoptosis in secondary SCI remains unclear. Therefore, we chose miR-129-5p for further investigation. Next, the expression change of miR-129-5p was calculated in spinal cord tissues of mice at different time points. As shown in Figure 1E, the miR-129-5p expression levels were reduced at different time points after SCI compared with the expression in the sham group. The decrease in level of expression of miR-129-5p was minimal at 7 days post-injury and then its expression was gradually increased until 28 days after injury. All data indicates that miR-129-5p may be involved in the pathogenesis of SCI.

### Agomir-miR-129-5p injection improved functional recovery and suppressed neurons apoptosis

**Figure 2:**
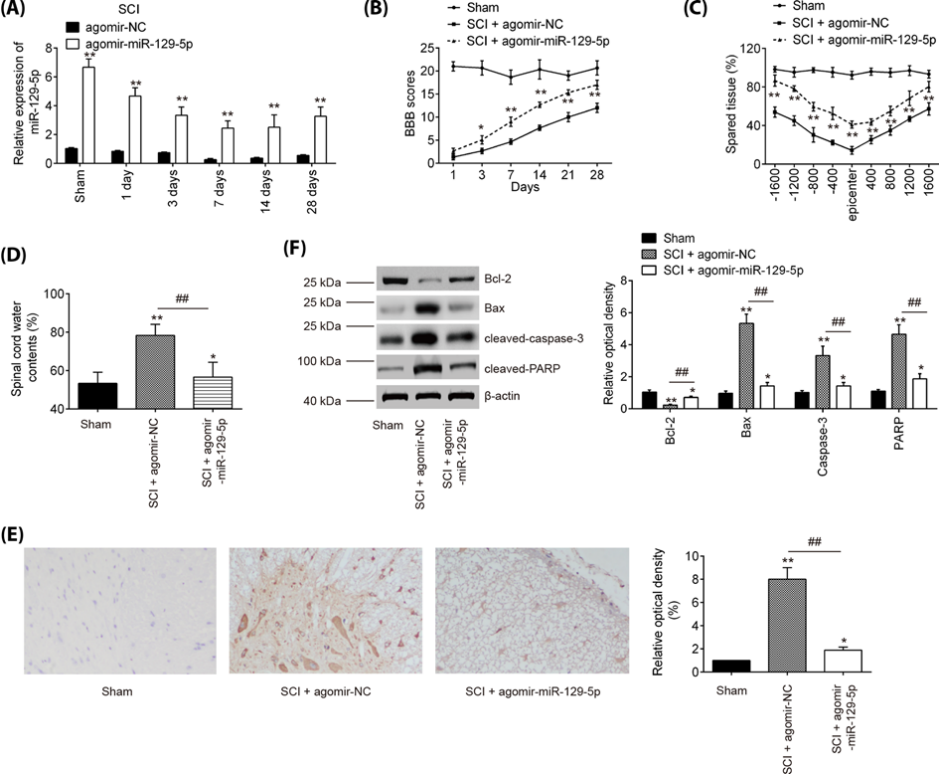
Agomir-miR-129-5p improves recovery of SCI mice by reducing apoptosis The mice were subjected to SCI and then treated with agomir-miR-129-5p/agomir-NC (50 μl/day, 100 nmol/ml) for 3 days (0, 1, and 2 days) via intrathecal injection starting 15 min after contusion SCI. At indicated time, the animals were killed and subsequently, a 10-mm long segment of the spinal cord was harvested for further experiments. (**A**) qRT-PCR was performed to determine the expression levels of miR-129-5p in spinal cord tissues at 1, 3, 7, 14, and 28 days after agomir-129-5p injection. (**B**) The BBB scores at 1, 3, 7, 14, 21, and 28 days after SCI were shown for all groups of mice. (**C**) Cresyl Violet staining was used to assess spared tissue following behavioral analyses at 28 days post-injury. (**D**) Spinal cord water content was assessed using wet-to-dry weight method. (**E**) IHC analysis of cleaved caspase-3 at 7 days post-injury in spinal cord tissues. (**F**) The protein expressions of Bcl-2, Bax, cleaved caspase-3 and cleaved PARP were measured by Western blot. Data represent the mean ± SD of three independent experiments. *P<0.05, **P<0.01 vs. Sham group; ^##^*P*<0.01 vs. SCI + agomir-NC group.

To examine the impact of miR-129-5p in SCI, agomir-miR-129-5p and agomiR-NC were intrathecally injected into SCI mice. miR-129-5p expression level was significantly increased in spinal cord of SCI mice from 1 to 28 days post-injury, compared with agomiR-NC group (Figure 2A). Subsequently, the hindlimb motor function recovery of SCI mice was evaluated using BBB score. Agomir-miR-129-5p injection indeed improved functional recovery from 3 days compared with that in the agomir-NC injection group (Figure 2B). Using Cresyl Violet staining assay, it was found that the amount of spared tissue was markedly increased in SCI + agomir-miR-129-5p group compared with the agomir-NC-treated SCI mice, indicating that agomir-miR-129-5p can reduce lesion size in SCI mice (Figure 2C). Meanwhile, the water contents in spinal cord were found to be dramatically decreased after administering with agomir-miR-129-5p, compared with the agomir-NC-treated SCI mice group (Figure 2D), suggesting the alleviation of spinal cord edema. To test whether apoptosis accounted for the protection of miR-129-5p against SCI, the cleaved-caspase-3 expression was measured by immunohistochemistry staining. As shown in Figure 2E, SCI resulted in a significant increase in the expression of cleaved-caspase-3 compared with Sham group, while agomir-miR-129-5p significantly reduced SCI induced expression of cleaved-caspase-3 in spinal cord tissues. Furthermore, Western blot was used to detect the expression of Bcl-2, Bax, cleaved-caspase-3, and cleaved-PARP in spinal cord tissues following agomir-miR-129-5p treatment. As expected, agomir-miR-129-5p markedly increased the expression level of Bcl-2, and decreased the expression levels of Bax, cleaved-caspase-3, and cleaved-PARP compared with agomir-NC-treated SCI mice (Figure 2F). Taken together, miR-129-5p up-regulation could promote functional recovery and protect neurons against SCI-induced apoptosis in mice.

### Agomir-miR-129-5p injection inhibited the inflammatory response in SCI mice

**Figure 3:**
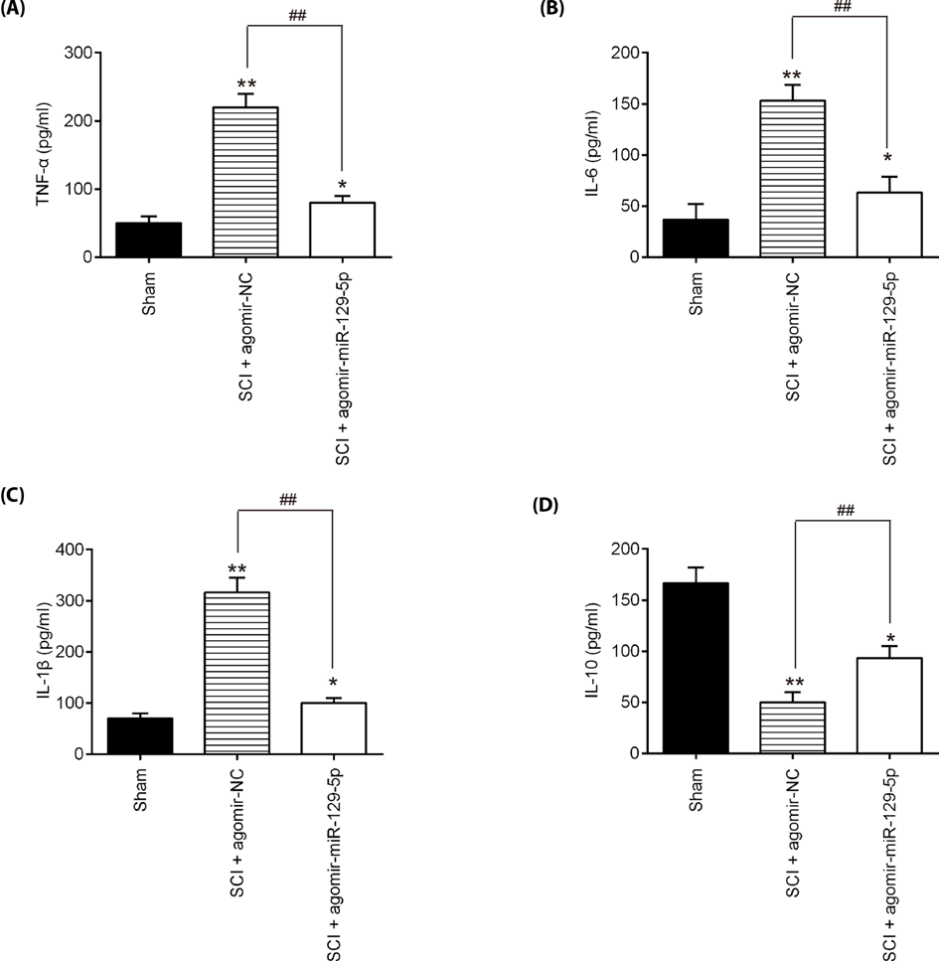
miR-129-5p inhibited the inflammatory response in SCI mice The mice were subjected to SCI and then treated with agomir-miR-129-5p/agomir-NC (50 μl/day, 100 nmol/ml) for 3 days (0, 1 and 2 days) via intrathecal injection starting 15 min after SCI. At indicated time, the animals were killed, and subsequently serum samples were collected for detection of production of inflammatory factors. The levels of TNF-α (**A**), IL-6 (**B**), IL-1β (**C**) and IL-10 (**D**) in serum of mice were measured by ELISA kits. Data represent the mean ± SD of three independent experiments. *P<0.05, **P<0.01 vs. Sham group; ^##^*P*<0.01 vs. SCI + agomir-NC group.

To further evaluate the influence of antagomiR-129-5p on the inflammatory response, the releases of TNF-α, IL-6, IL-1β, and IL-10 in serums of SCI mice were detected by ELISA. As shown in Figure 3A–D, the expression levels of TNF-α, IL-6, IL-1β were significantly increased, and IL-10 was markedly decreased compared with the Sham group. However, miR-129-5p up-regulation significantly reduced the expressions of these pro-inflammatory cytokines, while enhanced the levels of IL-10 induced by SCI. These data indicated that agomir-miR-129-5p suppressed the SCI-induced inflammatory response in mice.

### Overexpression of miR-129-5p suppressed apoptosis and inflammatory response in LPS-treated BV-2 cells

**Figure 4:**
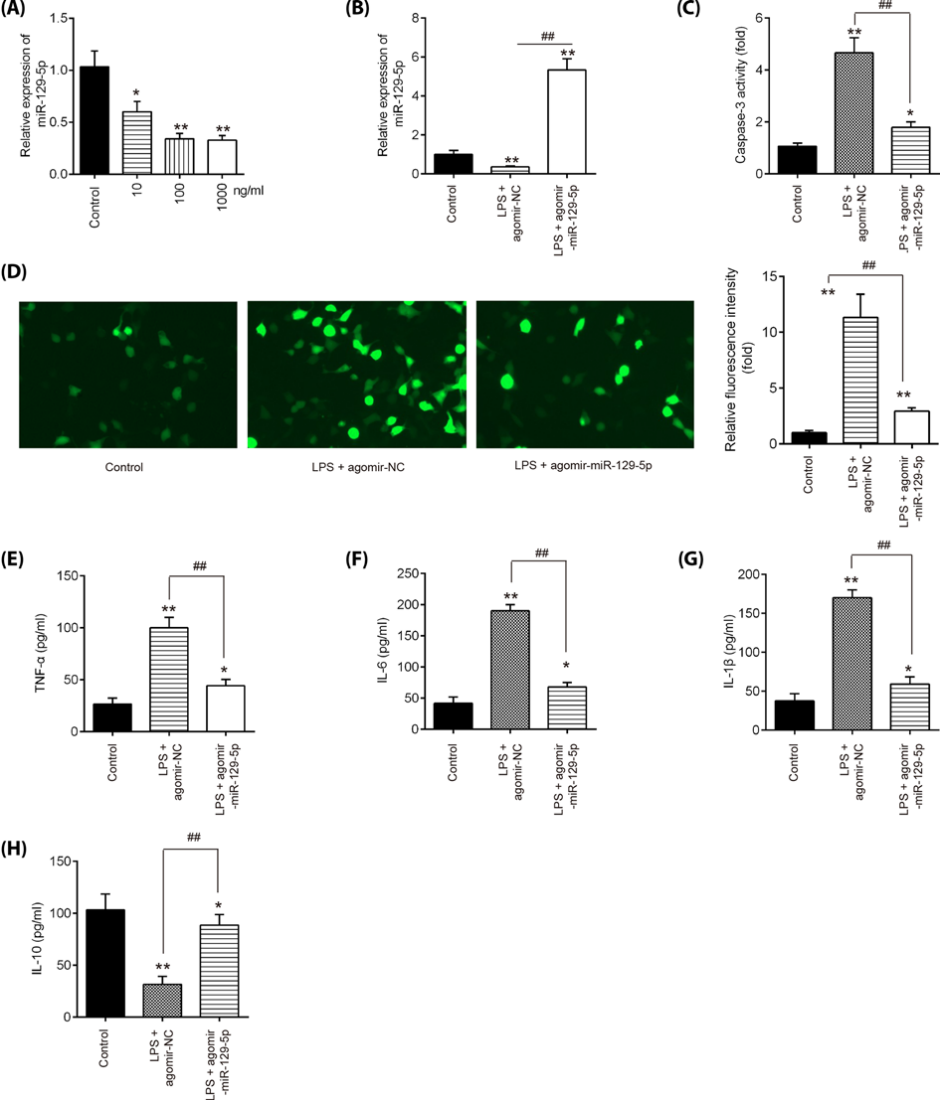
Overexpression of miR-129-5p suppressed inflammatory response and apoptosis in SCI cell model (**A**) BV-2 cells were treated with different concentrations of LPS (10, 100, and 1000 ng/ml) for 24 h, and the expression of miR-129-5p was detected by qRT-PCR analysis. (**B**) Agomir-miR-129-5p was added to the cultured BV-2 cells 4 h prior to LPS treatment and incubated for 24 h, and then the transfected efficiency of agomir-miR-129-5p was detected by qRT-PCR analysis. (**C**) Activity of caspase-3 was measured using a caspase-3 activity assay kit. (**D**) The protein expression level of caspase-3 in BV-2 cells was detected by IFA. (**E**–**H**) The expressions of TNF-α, IL-6, IL-1β, and IL-10, were measured by ELISA analysis. Data represent the mean ± SD of three independent experiments. *P<0.05, **P<0.01 vs. Control group; ^##^*P*<0.01 vs. LPS + agomir-NC group.

In order to explore the molecular mechanism involved in the protection of miR-129-5p in secondary SCI-induced apoptosis and inflammatory response, we established n SCI cell model using LPS-treated BV-2 cells as previously described [36]. Following various concentrations of LPS (10–1000 ng/ml) treatment, miR-129-5p was significantly down-regulated compared with control group in BV-2 cells (Figure 4A). And no differences were detected between the two groups of cells treated with 100 and 1000 ng LPS. Thus, 100 ng/ml LPS was selected as the appropriate concentration in the subsequent experiments, which is also consistent with a previous study [37]. To investigate the functions of miR-129-5p in LPS-induced BV-2 cell injury, the agomir-miR-129-5p was added to BV-2 cells 4 h prior to LPS treatment. qRT-PCR assay showed that miR-129-5p was notably increased after agomir-miR-129-5p treatment in BV-2 cells (Figure 4B). Functional experiments revealed that agomir-miR-129-5p markedly reduced LPS induced the activity of caspase-3 and the expression of caspase-3, determined as using caspase-3 activity assay and IFA assay in BV-2 cells, respectively (Figure 4C,D). Additionally, the impact of miR-129-5p on the expression levels of inflammatory cytokines was further assayed. As expected, agomir-miR-129-5p treatment markedly inhibited the expression levels of TNF-α, IL-6, IL-1β, but promoted the expression of IL-10 in LPS plus agomir-miR-129-5p, compared with LPS plus agomir-NC group (Figure 4E–H). All data suggest that miR-129-5p up-regulation improved the LPS-induced BV-2 cell injury.

### HMGB1 is a direct target of miR-129-5p in BV-2 cells

**Figure 5:**
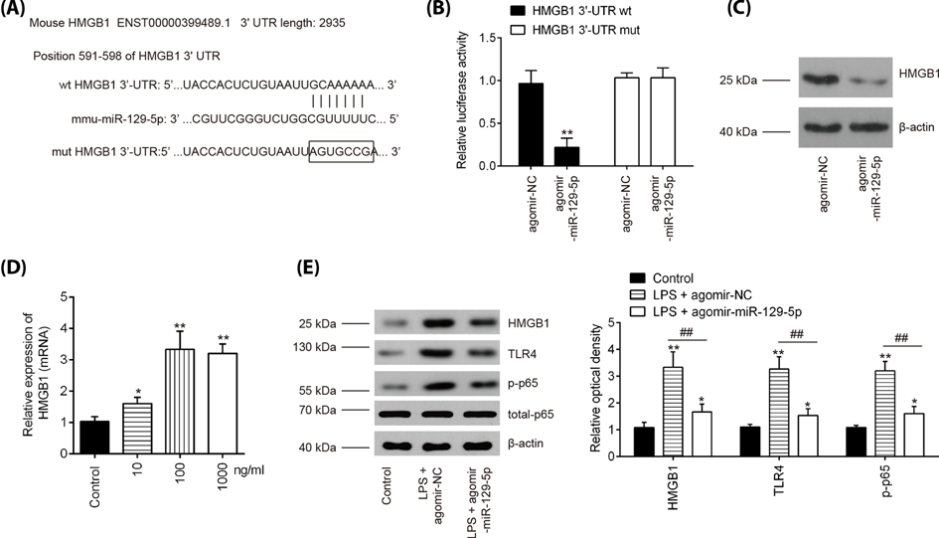
HMGB1 is a direct target of miR-129-5p in BV-2 cells (**A**) Putative binding site of miR-129-5p and HMGB1 with mut and wt 3′UTRs. (**B**) Luciferase assay of BV-2 cells co-transfected with firefly luciferase constructs containing the HMGB1 wild-type or mutated 3′-UTRs and agomir-miR-129-5p or agomir-NC, as indicated (*n*=3). Data represent the mean ± SD of three independent experiments. **P<0.01 vs. agomir-NC group. (**C**) The protein levels of HMGB1 were detected by Western blot after agomir-miR-129-5p transfection. (**D**) BV-2 cells were treated with different concentrations of LPS (10, 100 and 1000 ng/ml) for 24 h, and the expression of HMGB1 was detected by qRT-PCR analysis. (**E**) The protein expression levels of HMGB1, TLR4, p-p65 and total p65 were detected by Western blot analysis. Data represent the mean ± SD of three independent experiments. *P<0.05, **P<0.01 vs. Control group; ^##^*P*<0.01 vs. LPS + agomir-NC group.

To explore the potential mechanisms in which miR-129-5p protected BV-2 cells against LPS- apoptosis and inflammatory response, we performed TargetScan 7.0 and miRanda to predict the targets of miR-129-5p. Bioinformatics analysis indicated that HMGB1 was a potential target of miR-129-5p (Figure 5A). Previous studies have reported that HMGB1 plays an amplifying role in tissue pathology and inflammation, leading to secondary damage after the initial SCI [38,39]. Thus, we chose it for next study. Next, luciferase reporter assay was then performed in BV-2 cells to determine whether miR-129-5p directly targets HMGB1. As shown in Figure 5B, agomir-miR-129-5p significantly repressed the luciferase activity of the HMGB1-3′UTR wt reporter plasmid, but not that of the mutant reporter. Furthermore, the results of Western blot analysis showed that agomir-miR-129-5p notably reduced protein levels of HMGB1 in BV-2 cells (Figure 5C). It was also observed that the mRNA levels of HMGB1 were significantly up-regulated in BV-2 cells following LPS challenge (Figure 5D), which is consistent with a previous study [40].

HMGB1 signaling through multiple receptors, such as TLR4 promotes activation of the NF-κB transcription factor, which is directly related to inflammatory response in secondary SCI [39,41]. Further studies were designed to examine the influence of miR-129-5p on the activation of TLR4/NF-κB *in vitro*. The results showed that LPS induced the expressions of HMGB1, TLR4, and p-p65 at protein level, whereas agomir-miR-129-5p overexpression reversed these promoting effects of LPS in BV-2 cells (Figure 5E). All these results indicated that the miR-129-5p inhibited the inflammation via HMGB1/TLR/NF-κB pathway.

### miR-129-5p protects BV-2 cells from LPS-induced apoptosis and inflammatory response by targeting HMGB1

**Figure 6:**
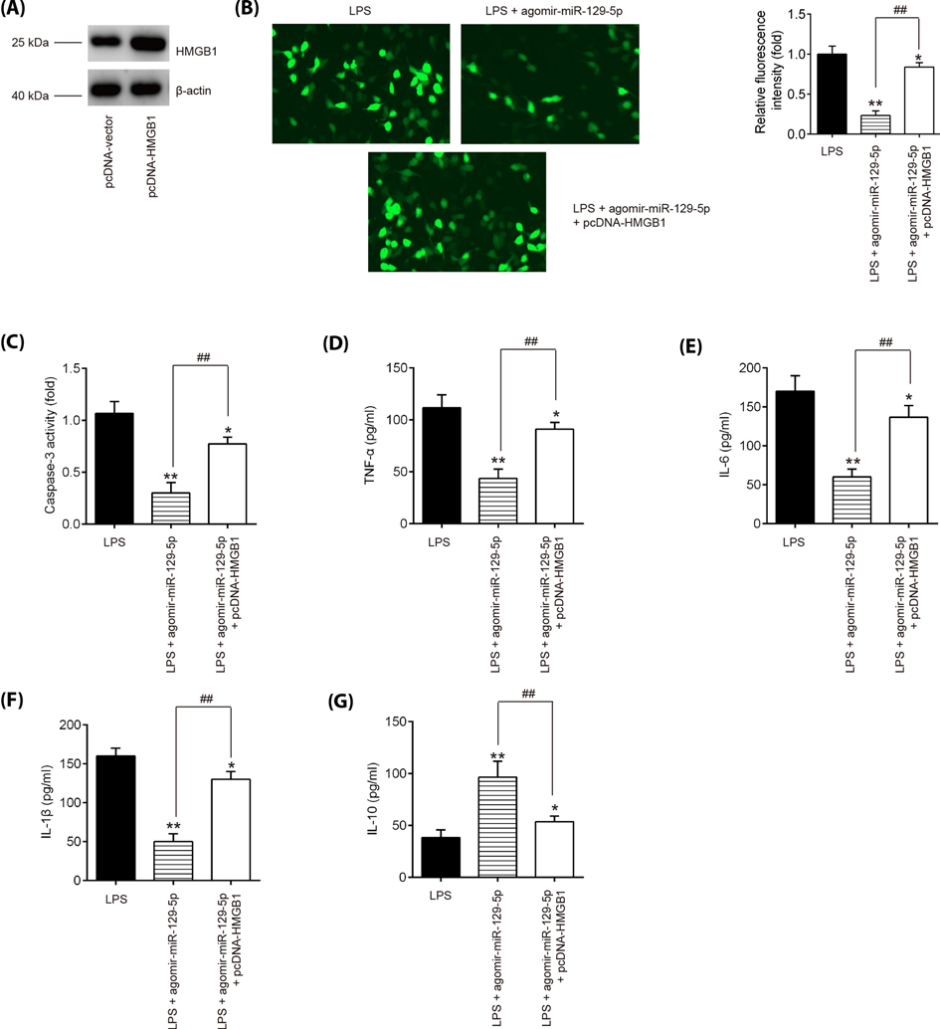
miR-129-5p protects BV-2 cells from LPS-induced apoptosis and inflammatory response by targeting HMGB1 Agomir-miR-129-5p and pcDNA-HMGB1 were co-transfected into the cultured BV-2 cells 4 h prior to LPS treatment, and incubated for 24 h, then cells were harvested for next experiments. (**A**) The transfected efficiency of pcDNA-HMGB1 was determined by Western blot. (**B**) The protein expression level of caspase-3 was detected by IFA in BV-2 cells. (**C**) Activity of caspase-3 was measured using a commercial kit. (**D**–**G**) The expressions of TNF-α, IL-6, IL-1β, and IL-10 were measured by ELISA analysis. Data represent the mean ± SD of three independent experiments. *P<0.05, **P<0.01 vs. LPS group; ^##^*P*<0.01 vs. LPS + agomir-miR-129-5p group.

Given the indispensable role of HMGB1 in the inflammatory response following SCI, we tried to determine whether HMGB1 mediated the protective effects of miR-129-5p on the inflammation in SCI. We transfected HMGB1 expression vector, pcDNA-HMGB1, together with agomir-miR-129-5p into BV-2 cells 4 h prior to LPS treatment. It was found that the protein expression of HMGB1 was significantly increased after pcDNA-HMGB1 transfection in BV-2 cells (Figure 6A). Functionally, agomir-miR-129-5p treatment inhibited the caspase-3 expression and the caspase-3 activity in LPS-treated BV-2 cells, whereas overexpression of HMGB1 by pcDNA-HMGB1 attenuated the inhibitory effect of miR-129-5p on the protein expression of caspase-3 and caspase-3 activity (Figure 6B,C). Furthermore, inflammatory cytokine productions were evaluated using ELISA in LPS-treated BV-2 cells following pcDNA-HMGB1 and agomir-miR-129-5p co-transfection. As shown in Figure 6D–G, agomir-miR-129-5p treatment markedly decreased TNF-α, IL-6, IL-1β protein expression levels and increased the IL-10 expression in LPS-treated BV-2 cells, while these effects of agomir-miR-129-5p was reversed by overexpression of HMGB1. These data suggest that miR-129-5p protects BV-2 cells from LPS-induced apoptosis and inflammatory response by targeting HMGB1, indicating agomiR-129-5p may improve secondary SCI through HMGB1/TLR4/NF-κB pathway.

## Discussion

In the present study, miR-129-5p was found to be significantly down-regulated in spinal cord tissues of SCI mice and LPS-induced BV-2 cell injury model. Agomir-miR-129-5p injection improved the functional recovery and reduced the spared tissues and water content of spinal cord in SCI mice. Moreover, miR-129-5p overexpression inhibited SCI induced inflammatory response *in vivo* and *in vitro*. Notably, the data indicated that the overexpression of miR-129-5p may exert protective effects by blocking HMGB1/TLR4/NF-κB pathway activation. Our findings could provide a new guidance for the improvement of SCI patients in the future.

A number of studies demonstrated that miRNAs are aberrantly expressed in SCI, and may influence secondary SCI pathophysiology, such as inflammation and apoptosis [24,42,43]. For example, Xu et al. found that miR-124 improved functional recovery and suppressed neuronal cell apoptosis by blocking the mitochondrial apoptotic pathway in SCI rats [44]. Feng et al. found that miR-204-5p level in the SCI mice was decreased, and overexpression of miR-204-5p restored upper and lower limb strength of mice by suppressing inflammation below the injury site [24]. Another study performed by Xu et al. reported that miR-124 may improve functional recovery and suppress neuronal cell apoptosis by blocking the mitochondrial apoptotic pathway in SCI mice [44]. In the present study, using an miRNA microarray, we found large numbers of miRNAs were significantly deregulated; in particular, miR-129-5p was identified as the most down-regulated miRNA in spinal cord tissues from SCI mice, suggesting miR-129-5p may be involved in secondary injury.

Several studies have shown that miR-129-5p acts as a novel regulator of the inflammatory response and apoptosis in various inflammatory diseases. For example, Li et al. reported that miR-129-5p suppressed the spinal cord ischemia–reperfusion (IR) injury-induced inflammation in mice by inhibiting HMGB1 and the TLR3-cytokine pathway [35]. Notably, Zou et al. showed that miR-129-5p overexpression inhibited the inflammation and apoptosis in palmitic acid (PA)-induced cardiomyocyte injury model [45]. As we known, apoptosis is another key process that influences the development of neuronal tissue damage following SCI [13]. Therefore, it was hypothesized that miR-129-5p may affect the secondary injury through the regulation of inflammation and apoptosis. In our study, we found that agomir-miR-129-5p injection could improve the functional recovery, reduced spared tissue and edema of spinal cord, and suppressed the inflammatory response and apoptosis in mice, indicating the miR-129-5p has a protection effect in secondary SCI by inhibiting neuronal cell inflammation and apoptosis. However, the underlying molecular mechanisms involved in miR-129-5p-mediated inflammation and apoptosis suppression have not been completely clarified.

HMGB1 is a well-known regulator in inflammatory responses [46,47]. It can be actively secreted from reactive astrocytes and microglia after pathogenic insult or tissue injury [48]. Extracellular HMGB1 triggers inflammatory responses through the activation of multiple receptors, such as TLRs in immune-competent cells, neurons, and astrocytes [49,50]. HMGB1 signaling through these receptors activated NF-κB transcription factor, which is required for the expression of the many mediators of inflammatory responses and cell survival, such as TNF-α, IL-1), and IL-6 [51]. HMGB1 has been shown to be up-regulated in several pre-clinical models of SCI, where it promotes the accruement of secondary injury [40]. Kang et al. showed that inhibition of HMGB1/TLR4/NF-κB signaling pathway improved the functional recovery of SCI rats [52]. Interestingly, several studies reported that miR-129-5p has a tumor-suppressive role by targeting HMBG1 in various human cancers [53,54]. Of note, Liu et al. showed that miR-129-5p suppressed the progress of autoimmune encephalomyelitis (AE)-related epilepsy by inhibiting HMGB1/TLR4/NF-κB pathway [55]. In our study, HMBG1 was proved to be a target of miR-129-5p in BV-2 cells and its expression was increased in LPS treated BV-2 cells. Moreover, our data showed that miR-129-5p reduced the levels of key TLR4/NF-κB pathway proteins by suppressing HMGB1. Furthermore, we found that overexpression of HMGB1 reversed the inhibitory effects of miR-129-5p on LPS-induced inflammatory response and apoptosis in BV-2 cells. All data suggest that miR-129-5p suppressed HMGB1 to blocked the TLR4/NF-κB pathway activation and therefore repressed the LPS-induced inflammatory response and apoptosis.

In conclusion, our data showed that miR-129-5p improved secondary damage and facilitate functional recovery through inhibiting inflammatory responses via inactivation of the HMGB1/TLR4/NF-κB pathway in SCI mice model. Our findings may provide a novel direction for the therapeutics of SCI.

## Abbreviations

Bax: BCL2-Associated X protein
BBB: Basso, Beattie and Bresnahan
Bcl-2: B-cell lymphoma-2
HMGB1: high-mobility group protein B1
IFN: interferons
IHC: immunohistochemical
IL: interleukin
i.p.: intraperitoneal
LPS: lipopolysaccharide
miRNA: microRNA
MyD88: myeloid differentiation factor 88
NC: negative control
NF-κB: nuclear factor-κB
OD: optic density
PARP: poly ADP-ribose polymerase
qRT-PCR: quantitative reverse transcription-PCR
RT: room temperature
SCI: spinal cord injury
SOX11: SRY-box containing gene 11
TLR: toll-like receptor
